# Exercise, Obesity and CNS Control of Metabolic Homeostasis: A Review

**DOI:** 10.3389/fphys.2018.00574

**Published:** 2018-05-17

**Authors:** John K. Smith

**Affiliations:** Departments of Academic Affairs and Biomedical Science, James H. Quillen College of Medicine, East Tennessee State University, Johnson City, TN, United States

**Keywords:** exercise, obesity, metabolic homeostasis, hypothalamus, ghrelin, BDNF, GLP-1, leptin

## Abstract

This review details the manner in which the central nervous system regulates metabolic homeostasis in normal weight and obese rodents and humans. It includes a review of the homeostatic contributions of neurons located in the hypothalamus, the midbrain and limbic structures, the pons and the medullary area postrema, nucleus tractus solitarius, and vagus nucleus, and details how these brain regions respond to circulating levels of orexigenic hormones, such as ghrelin, and anorexigenic hormones, such as glucagon-like peptide 1 and leptin. It provides an insight as to how high intensity exercise may improve homeostatic control in overweight and obese subjects. Finally, it provides suggestions as to how further progress can be made in controlling the current pandemic of obesity and diabetes.

## Introduction

According to the World Health Organization, non-communicable diseases are the leading cause of death worldwide, causing 39.5 million deaths (70% of total) in 2015; ischemic heart disease was the leading cause of mortality, accounting for 17.7 million or 45% of deaths. These statistics have been linked to a concomitant worldwide increase in the prevalence of obesity-associated disorders (in 2010, 18% of children and adolescents and 39% of adults worldwide were overweight or obese), most particularly type 2 diabetes mellitus (Chen et al., 2011; Global Health Estimates, 2015). This pandemic of nutrition-related non-communicable diseases (NR-NCD) is most striking in developing countries in which the rapid spread of supermarkets has contributed to an increased consumption of processed foods, saturated and total fats, sugar, and high caloric beverages (Demmier et al., 2017).

The current pandemic of NR-NCD has occurred in the setting a global reduction in physical activity. According to the World Health Organization, insufficient physical activity was one of the 10 leading risk factors for mortality, contributing to some 3.2 million deaths per year, and 69.3 million disability-adjusted life years in 2010. Globally, 20% of men, 27% of women, and 78% of boys and 84% of girls aged 11–17 did not meet recommendations for exercise health (Global Health Estimates, 2015). The World Health Organization recommends that adult men and women should accumulate at least 150 min of moderate intensity physical activity per week and young people aged 5–17 years should accumulate at least 60 min of physical activity of moderate to vigorous intensity daily (Global Health Estimates, 2015). Of note is that exercise done by young children is much more likely to protect against adult onset obesity than exercise regimens initiated later in life (Caruso et al., 2013), and obese children are likely to become obese adults, whereas children with a healthy weight have less than a 50% chance of becoming obese by the age of 35 (Ward et al., 2017).

Although the scientific community is aware of life style changes that can reduce the prevalence of NR-NCD (Laslett et al., 2012), its ability to invoke non-surgical measures that lead to permanent (lifelong) reductions in weight is lacking (Merlotti et al., 2014). The challenges are considerable since, as noted by Clemmensen and associates, “modern humans have (mis)used the legacy of superior brain-power to engineer a dietary environment that supersedes peripherally derived satiation and adiposity signals, exploits the limbic system, is “unnaturally” energy-dense and hyper-palatable, and comes in virtually unlimited quantities” (Clemmensen et al., 2017).

This review is intended to provide a better understanding of the homeostatic, non-homeostatic, and hedonic nervous system pathways that contribute to obesity and its associated co-morbidities, and how physical exercise may have a salutary effect when these pathways go awry.

## Materials and methods

This best evidence review is on the effect of physical exercise on the maintenance of metabolic homeostasis. The research strategy included: 1. Defining the key topics; 2. Identifying key words or synonyms that represent each of the key topics; 3. An online literature search of key topics and key words; and 4. A refinement of the search based on initial findings. Key topics included: global health statistics; exercise; metabolic homeostasis; obesity; diabetes mellitus; hypothalamus; orexigenic neurons, hormones and peptides; anorexigenic neurons, hormones and peptides; melanocortin system; arcuate nucleus; ventromedial nucleus; mesoaccumbens dopamine system; parabrachial nucleus; area postrema; nucleus tractus solitarius; vagus; gut-brain crosstalk; bariatric surgery; olfaction. Keywords included: exercise, obesity, metabolic homeostasis, hypothalamus, amygdala, area postrema, NTS, orexigenic, anorexigenic, ghrelin, BDNF, GLP-1, GDF15, leptin, melanocortin, NTS, POMC/CART, AgRP/NPY, vagus, bariatric surgery.

## CNS control of metabolic homeostasis

### The hypothalamus

The hypothalamus is the principle regulator of the autonomic nervous system, the seasonal, and circadian clock for behavioral and sleep-wake functions, the neural center of the endocrine system, and the primary regulator of thirst and hunger. It effects these functions by communicating with other control centers in the central and peripheral nervous systems, including the prefrontal and insular cortices, the amygdala and other limbic structures, the midbrain, the pons, the medulla, and the vagus and glossopharyngeal nerves. Of particular importance is the amygdaloid, whose central nucleus is a major site of origin of projections to the hypothalamus and brainstem.

### Arcuate and ventromedial hypothalamic nuclei

Located in the medial basal hypothalamus is the arcuate nucleus which plays an essential role in the regulation of food intake and energy expenditure (Myers and Olson, 2012; Williams, 2012; Joly-Amado et al., 2014). This nucleus contains orexigenic neurons that co-express agouti-related protein and neuropeptide Y (AgRP/NPY neurons) and anorexigenic neurons co-expressing pro-opiomelanocortin and cocaine and amphetamine-regulated transcripts (POMC/CART neurons). Unlike other hypothalamic nuclei, the arcuate nucleus is located outside the blood-brain barrier and thus is ideally situated to receive input from circulating nutrients and hormones; it also receives signals emanating from cerebrospinal fluid bathing the third ventricle (Williams, 2012).

AgRP/NPY expressing neurons enhance appetite and weight gain by γ-aminobutyric acid (GABA)-mediated tonic inhibition of POMC/CART neurons including the suppression of anorexigenic signals from neurons located in the parabrachial nucleus of the pons. The anorexigenic effects of the parabrachial nucleus come from glutaminergic signals originating in the nucleus tractus solitarius in the medulla which services the vagus nerve (Wu et al., 2012). Both insulin and leptin suppress appetite and weight gain by inhibiting AgRP/NPY neurons, and insulin action on these neurons suppresses hepatic gluconeogenesis, contributing to glycemic control (Myers and Olson, 2012). The orexigenic hormone ghrelin, which is produced by endocrine cells in the gastric mucosa, enhances food intake by activating AgRP/NPY neurons (Mason et al., 2014). Ghrelin also regulates synaptogenesis between AgRP/NPY and POMC/CART neurons (Williams, 2012). In experimental animals, obliteration of arcuate AgRP/NPY neurons results in loss of appetite, progressive weight loss and, eventually, death due to starvation (Carter et al., 2013).

POMC/CART expressing neurons produce α-melanocyte stimulating factor which on binding to melanocortin-3 (MC3R) and melanocortin-4 (MC4R) receptors promotes energy expenditure and suppresses food intake. MC3R are expressed at high levels in the ventromedial and arcuate nuclei, whereas MC4R are widely spread throughout the central nervous system, most notably in the ventromedial nucleus (Myers and Olson, 2012). POMC/CART neurons are inhibited by NPY and activated by leptin, serotonin, brain-derived neurotrophic factor (BDNF), and glial-cell-derived growth and differentiation neurotrophic factor 15 (GDF15), a divergent member of the transforming growth factor (TGF)-β superfamily (Williams et al., 2011; Macia et al., 2012; Spaeth et al., 2012; Fox et al., 2013; Tsai et al., 2014; Clemmensen et al., 2017). In experimental animals, obliteration of POMC/CART neurons in the ventromedial nucleus leads to morbid obesity (Macia et al., 2012).

Liu et al. provided evidence in mice that glucagon-like peptide-1 (GLP-1) expressing neurons in the medulla send anorexigenic signals to the paraventricular nucleus (PVN) of the hypothalamus and that postnatal depletion of GLP-1 receptors in the PVN causes hyperphagic obesity (Liu et al., 2017). GLP-1 is a potent incretin, and regulates glucose independent of its anorexigenic effects (Näslund et al., 2004).

Activation of the sympathetic nervous system by melanocortin receptors in the ventromedial nucleus enhances energy expenditure by increasing thermogenesis and fatty acid utilization in skeletal muscle and brown adipose tissue (Gavini et al., 2016). In this regard, neurons in the olfactory epithelium and bulb have a high density of receptors for insulin, leptin and ghrelin, and, because of an incomplete blood-brain barrier, the bulb has access to circulating levels of hormonal and nutrient signals in a manner similar to the arcuate nucleus; bulb signals are transmitted to the hypothalamus via pathways in the pyriform cortex and amygdala and increase energy expenditure by enhancing thermogenesis in brown adipose tissue. (Garrison and Knight, 2017).

### Area postrema, nucleus tractus solitarius, and vagus

The area postrema, a brainstem organ analogous to the hypothalamic arcuate nucleus, receives and integrates multiple metabolic signals, including anorexigenic signals from leptin, cholecystokinin, amylin, GLP-1, pancreatic peptide (PP) and peptide YY (PYY), and orexigenic signals from ghrelin (Wren, 2008; Clemmensen et al., 2017). This area projects to the nucleus tractus solitarius (NTS) which also receives continuous input from vagal afferent fibers located in the intestinal wall and gastric mucosa including those that relay information on the volume and composition of food being ingested (Clemmensen et al., 2017). As previously noted, the NTS relays these signals to the parabrachial nucleus in the pons. In turn, calcitonin gene-related peptide expressing neurons in the parabrachial nucleus send anorexigenic signals to the arcuate nucleus and central amygdala (Carter et al., 2013). Like the amygdala, the parabrachial nucleus helps place an emotional stamp on taste (e.g., taste aversion, taste preference). GLP-1 expressing neurons in the nucleus tractus solitarius relay anorexigenic signals to corticotrophin-releasing hormone (CRH), nefstatin-1, and oxytocin-expressing neurons in the paraventricular nucleus (Katsurada et al., 2014). GLP-1 receptor binding has been shown to restrict feeding by enhancing the strength of excitatory synaptic signals in paraventricular CRH neurons via a protein kinase A-dependent signaling cascade (Liu et al., 2017). The BDNF receptor TrkB is highly expressed in neurons in the area postrema, nucleus tractus solitarius, and dorsal motor nucleus of the vagus; evidence suggests that TrkB signaling in the NTS mediates the anorexigenic effects of brainstem BDNF (Spaeth et al., 2012).

The area postrema and NTS also contribute to non-homeostatic body weight regulation by expressing receptors for GDF15 (GDF15AL). GDF15LA ligation transmits anorexigenic signals to neurons located in the parabrachial and central amygdala nuclei. This pathway causes anorexia and weight loss in disorders associated with tissue stress or injury (Tsai et al., 2014; Hsu et al., 2017), and has been shown to decrease food intake, body weight, and improve glucose tolerance in mice on normal and obesogenic diets (Macia et al., 2012; Tsai et al., 2017). GDF15 is also known as macrophage inhibitory cytokine-1 (MIC-1) (Tsai et al., 2014).

By communicating with neurons in the NTS, vagal neurons expressing receptors for gut-derived hormones play a significant role in appetite regulation. GLP-1 binding induces an anorexigenic phenotype in afferent vagal neurons, an effect that is down-regulated by ghrelin (Ronveaux et al., 2015); and cholecystokinin-induced inhibition of food intake is dependent on signaling from vagal afferent neurons. Importantly, vagus afferent neurons can change their phenotype and express either orexigenic or anorexigenic receptors depending on the availability of nutrients (Ronveaux et al., 2015).

### Midbrain and limbic system

As noted, taste aversions and taste preferences are mediated in part by nuclei located in the central amygdala. However, food reward signals are mediated primarily by midbrain dopaminergic neurons that project rostrally to the nucleus accumbens (the mesoaccumbens dopamine system) and then to other areas in the limbic system, ultimately reaching the prefrontal cortex where conscious decisions regarding eating are made (Clemmensen et al., 2017). Importantly, growth hormone secretagogue receptors (GHSRs) in dopaminergic neurons mediate ghrelin-induced homeostatic feeding and ghrelin-engaged food-reward behavior. And GHSRs in the hippocampus and amygdala mediate more complex behaviors related to food intake, including cue-potentiated feeding (Mason et al., 2014).

## Obesity and metabolic homeostasis

### The hypothalamus

The ability of the hypothalamus to control energy balance is “compromised and degraded” in the majority of obese individuals (Williams, 2012). Aberrant functioning can be found at many levels, including the integration of signals of satiety and the regulation of glucose and lipid metabolism. In addition, there is a mechanistic link between over-eating, particularly of long chain fatty acids, and the occurrence of systemic inflammatory responses that can degrade the functioning of the hypothalamus in obese individuals (Arkan et al., 2005; Williams, 2012; de Git and Adan, 2015).

### Arcuate and ventromedial hypothalamic nuclei

#### Studies in rodents

In rats, high-fat, high-energy feeding has been shown to cause resistance to the anorexic and thermogenic effects of leptin due to reduced mRNA expression and transduction capabilities of the long form of the leptin receptor in arcuate nuclei (Patterson et al., 2009). High fat diets have also been shown to reduce synapses on POMC neurons (Horvath et al., 2010), and suppression of neurogenesis is reported to occur in the arcuate nuclei of mice with diet or leptin deficiency-induced obesity (McNay et al., 2012). Baver and associates have shown that diet-induced obesity in mice is associated with a failure of leptin to inhibit the orexigenic effects of AgRP neuronal activity (Baver et al., 2014).

#### Studies in humans

Humans with single nucleotide polymorphisms in the MC4R are morbidly obese, hyperphagic, and have elevated leptin levels and insulin resistance (Haskell-Luevano et al., 2009); those with deficiencies in the leptin or leptin receptor gene, or BDNF-related genetic polymorphisms also have hyperphagic obesity (Spaeth et al., 2012). Although blood levels of leptin increase in proportion to the triglyceride content of white adipose tissue, obese humans have reduced sensitivity to its anorexigenic effects; this resistance is incompletely understood but may be the consequence suppressor-of-cytokine-signaling (SOCS-3) blocking the central effects of leptin (Greenberg and Obin, 2006). Fasting ghrelin levels in children with the Prader-Willi syndrome are elevated and may play a role in their insatiable appetite whereas fasting levels in both normal and obese individuals are inversely related to their body mass index (Haqq et al., 2003). And serum levels of BDNF are reported to be depressed in obese subjects with type 2 diabetes (Li et al., 2016).

### Area postrema, nucleus tractus solitarius, parabrachial nucleus, and vagus

#### Studies in rodents

In mice, high-fat, high-energy feeding is reported to impair anorexigenic signals in the caudomedial nucleus of the NTS (Cavanaugh et al., 2015), and to alter circadian expression of molecular clock genes in this nucleus (Kaneko et al., 2009). Treatment with GDF15, which binds to neurons in the NTS, the area postrema, and the arcuate nucleus, has been shown to decrease adiposity and correct metabolic function in diet-induced obese mice (Tsai et al., 2017).

Zhang and associates found that pro-opiomelanocortin gene transfer to the NTS but not to the arcuate nucleus ameliorates diet-induced obesity in rats, emphasizing the critical role that the NTS plays in energy regulation (Zhang et al., 2010). And Kovacs and Hajnal found that obese rats lacking cholecystokinine-1 receptors had fewer sucrose-responsive neurons in the parabrachial nucleus and an overall reduction in taste response magnitude to sucrose when compared to lean animals (Kovacs and Hajnal, 2008).

Administration of native GLP-1 and GLP-1 agonists such as exendin-4 and liraglutide have been shown to reduce food intake and weight in obese rodents by inducing anorexigenic phenotype switching in afferent vagal neurons; unlike its analogs, native GLP-1is rapidly degraded in the circulation, limiting its potential as a therapeutic agent (Ronveaux et al., 2015).

#### Studies in humans

Plasma GLP-1 levels are reported to be low in obese individuals with normal and impaired glucose tolerance (Hussein et al., 2014), and administration of native GLP-1 and GLP-1 agonists have been shown to reduce food intake and weight in obese humans (Näslund et al., 2004). GLP-1 is the most powerful known incretin (i.e., insulin releaser) in humans and serves as the basis for recent treatments of diabetes. Acute high intensity exercise has been shown to increase GLP-1 levels and reduce hunger scores in obese subjects, although the rise was less than that seen in normal weight subjects (Ueda et al., 2013).

In his review of the therapeutic effects of obesity surgery, Blasi notes the rapid and often persistent remission of type 2 diabetes and the metabolic syndrome following bariatric surgery, and reviews the major role that the vagal NTS and vaso-vagal pathways play in restoring normal function to the pancreas (normalizing insulin secretion, reducing glucagon production), liver (recovering insulin sensitivity, reducing gluconeogenesis and free fatty acid release), and gastrointestinal tract (reducing ghrelin secretion, restoring normal responses to nutrients, peptides, hormones) (Blasi, 2016).

### Midbrain and limbic system

#### Studies in rodents

The inactivity characteristic of obesity is associated with reductions in dopamine receptor signaling in the mesoaccumbens dopamine system, most notably from D2R^+^ neurons in the nucleus accumbens (Zhu et al., 2016; Ruegsegger and Booth, 2017). Voluntary wheel running in obese rats is reported to attenuate the metabolic syndrome in MC4R deficient rats with dopamine dysregulation even though the reward signals are blunted; MC4R are highly expressed by dopamine secreting neurons in the mesolimbic dopamine pathway (Obici et al., 2015).

Mice rendered hyposmic by ablation of olfactory nerves have been shown to be resistant to diet-induced obesity; this has been attributed to an increase in heat dissipation by brown adipose tissue. In contrast, mice bred to have an increased sense of smell increase their body weight in the absence of any change in food intake (Riera et al., 2017). These studies indicate that the olfactory system can regulate body weight by direct effects on energy expenditure rather than solely through changes in food intake. Surgically bypassing the duodenum and jejunum in mice has been shown to reduce the dopamine-stimulating and appetite enhancing effects of intragastric glucose.

#### Studies in humans

Hedonic eating is the consumption of palatable foods beyond the need-based energy requirements of the organism and is prevalent in overweight and obese individuals (Clemmensen et al., 2017). The remarkable success of gastric bypass surgeries in controlling overweight has been attributed, in part, to a reduction in hedonic eating. Studies in humans have shown that Roux-en-Y gastric bypass patients are less preoccupied with eating and begin to prefer low calorie over high calorie more palatable foods (Ernest et al., 2009; Schultes et al., 2010; Ullrich et al., 2013).

## Exercise and metabolic homeostasis

### Arcuate and ventromedial hypothalamic nuclei

#### Studies in rodents

Eight-weeks of voluntary exercise has been shown to prevent obesity, hyperphagia, hyperleptinemia and glucose intolerance in young MC4R knockout mice; paradoxically, this effect was associated with increased NPY and decreased POMC and MC3R expression in arcuate nuclei (Haskell-Luevano et al., 2009). Others report similar findings in obese rodents who lost weight and had blunted appetites despite having elevated hypothalamic NPY levels (Bi et al., 2005; Kawaguchi et al., 2005; Wang et al., 2008). Although hypothalamic levels of NPY can be expected to increase in energy-deficient, weight loss (≥ 30%), intensely exercising and food restricted rats, such was not the case in these studies, indicating that other factor(s) have overridden the orexigenic effects of NPY. In this regard, Kawaguchi and associates found that exercise-related weight loss in rats was associated with both an increase in arcuate nuclei NPY and corticotropin-releasing factor (CRF) expression in dorsomedial nuclei; intracerebro-ventricular injection of a CRF antagonist attenuated the weight loss, suggesting that CRF was the cause of the exercise-associate anorexia (Kawaguchi et al., 2005). And Wang and associates measured hypothalamic concentrations of NPY and ghrelin in diet-induced obese rats after short-term and long-term treadmill exercises and found that although NPY hypothalamic concentrations increased following both exercise regimens, hypothalamic ghrelin levels fell. The authors concluded that the weight loss and reduced appetite seen in their exercising rats was due to reduced hypothalamic levels of ghrelin (Wang et al., 2008).

In leptin deficient mice and diet-induced obese rats, 1 h of swimming and treadmill running has been shown to suppress hyperphagia and restore total energy intake, in part by returning hypothalamic POMC mRNA levels to normal. Exercise also increased serum and hypothalamic levels of interleukin (IL)-6; when injected into the third ventricle of obese rats, IL-6 reduced food intake and restored the anorexigenic effects of insulin and leptin by promoting IL-10-mediated inhibition of I_K_B kinase β/NF-_K_B signaling and ER stress responses (Ropelle et al., 2010). Transforming growth factor (TGF)-β, another antiinflammatory cytokine, has been shown to enhance fat oxidation by activating noradrenergic neurons in the ventromedial and paraventricular hypothalamic nuclei (Fujikawa et al., 2007).

Other studies have shown the beneficial effects of exercise on CNS responses to leptin or insulin. Krawczewski and co-authors found that voluntary exercise in a mouse strain susceptible to diet-induced obesity decreased fat mass & increased energy expenditure via activation of leptin receptor-positive neurons in the ventromedial nucleus; in this study, intracerebral leptin did not decrease body weight or food intake in sedentary mice fed high fat diets but did reduce body weight in exercising mice. They concluded that exercise leads to the maintenance of a lower body weight and leaner composition by improving CNS leptin action in obese animals (Krawczewski Carhuatanta et al., 2011). Chiarreotto-Ropelle and co-authors found that exercise improved insulin and leptin signaling in obese rats by disrupting the interaction between hypothalamic protein tyrosine kinase phosphatase B with proteins involved in the early steps of insulin and leptin signaling (Chiarreotto-Ropelle et al., 2013) and Patterson and associates found that postweaning exercise in diet induced obese rats produced prolonged increases in central leptin sensitivity and signaling (Patterson et al., 2009). In this regard, Caruso and coauthors found that a short period of exercise early in life had lasting beneficial effects on body weight, adiposity and hormone profile of rats from obese mothers despite being followed by a period of inactivity (Caruso et al., 2013). Exercise has also been shown to increase the expression of BDNF in rodents (Araya et al., 2013).

#### Studies in humans

High intensity exercise has been shown to reduce plasma levels of ghrelin and increase levels of GLP-1, BDNF, and GDF15; as previously noted, ghrelin exerts its orexigenic effects in part by activating AgRP/NPY neurons, and BDNF and GDF15 exert some of their anorexigenic effects by activating the melanocortin pathway.

Holliday and Blannin documented transient decreases in ghrelin and increases in GLP-1in 12 endurance trained men following 15–45 min of cycling at ~76% VO_2mx_; there was no change in appetite, but relative energy intake (intake-expenditure) was reduced (Holliday and Blannin, 2017). Similar findings were reported by Douglas and associates in a study involving 15 healthy men who ran for 60 min on treadmills at ~70% VO_2mx_; exercise elicited a high level of energy expenditure (7,566 ± 635 kJ) but did not produce compensatory changes in appetite or energy intake (Douglas et al., 2015). Kojima and associates found that a 20-km run done by well-trained long-distance runners decreased plasma ghrelin levels and energy intake as compared to non-exercising controls (Kojima et al., 2016). Broom et al. conducted a study in which nine healthy men ran for 55 min at 52% peak oxygen uptake (VO_2peak_) or 36 min at 75% VO_2peak_ and another nine men ran for 45 or 90 min at 70% VO_2peak_; in both groups, plasma levels of acylated ghrelin and hunger ratings fell and remained suppressed for up to 1.5 h (Broom et al., 2017). Importantly, high intensity interval training (15 second sprints at 170% VO_2max_ with 60 second periods of active recovery at 32% VO_2max_) is reported to decrease ghrelin levels and energy intake in obese subjects (Sim et al., 2014).

Araya et al. found that 30 sessions of aerobic exercise done by 15 overweight subjects increased their serum BDNF levels (Araya et al., 2013). Galliera and associates found that high intensity exercise elevated circulating levels of GDF15 in elite rugby players, presumably by activating ROS and ER-stress pathways (Galliera et al., 2014). In a study involving seven healthy males exercising at 67% of their VO2max, Kleinert et al. found that plasma GDF15 levels increased 34% immediately after exercise and by 64% above resting values 120 min after cessation of exercise (Kleinert et al., 2018). And serum BDNF rose significantly in 14 elderly women following a 16-week aquarobic exercise program (Kim and Kim, 2018).

Although exercise reduces leptin levels in proportion to the triglyceride content of white adipose tissue in obese individuals (Leal-Cerrro et al., 1998), evidence in humans to support the role of exercise in reducing obesity-related leptin resistance is lacking.

### Area postrema, nucleus tractus solitarius, parabrachial nucleus, and vagus

#### Studies in rodents

Using immunochemical labeling of the protein product of the proto-oncogene *c-fos* in rat brains, Iwamoto, and associates found that treadmill exercise resulted in increased labeling (i.e., potential gene activation) of areas involved in autonomic nervous system and somatomotor control, including the parabrachial nucleus, the medial portion of the NTS, and medullary areas containing the area postrema (Iwamoto et al., 1996). Barna and associates found similar *c-fos* labeling of diencephalic and brainstem areas in exercising rats (Barna et al., 2012).

#### Studies in humans

As previously noted, high intensity exercise can reduce plasma levels of ghrelin and increase levels of BDNF and GDF15, all of which act on brainstem neurons to enhance (ghrelin) or reduce (BDNF, GDF15) food intake. High intensity exercise can also cause transient increases in plasma levels of the gut hormones GLP-1, PP, and PYY (Martins et al., 2007) whose appetite-suppressing effects are mediated by afferent vagal neurons. Importantly, GLP-1 binding induces an anorexigenic phenotype in afferent vagal neurons, an effect that is down-regulated by ghrelin (Ronveaux et al., 2015).

### Midbrain and limbic system

#### Studies in rodents

Activation of neurons expressing D1 and D2 dopamine receptors in the nucleus accumbens has been shown to regulate voluntary running, locomotion, and food intake in rodents (Zhu et al., 2016). And voluntary wheel running in exercise-habituated rats has been shown to be dependent on both the nucleus accumbens and the medial prefrontal cortex (Basso and Morrell, 2015). Chen et al. reported that moderate intensity treadmill exercise decreased the preference of diet-induced obese mice for high-fat diets as compared to non-exercising controls; they provided evidence that this change in food preference was associated with dopamine plasticity in the mesoaccumbens dopamine system (Chen et al., 2017).

#### Studies in humans

Panek and associates found that moderate intensity exercise done 3–5 days per week by previously inactive human subjects reduced the reinforcing value (motivation to eat) of high energy density foods, although it had no effect on food preferences. It is not known whether ghrelin, which binds to GHSRs in the amygdala and dopaminergic neurons, played a role in this effect (Panek et al., 2014).

## Summary points

1. Leptin is produced in adipocytes and reduces appetite and weight gain by inhibiting AgRP/NPY neurons and stimulating POMC/CART neurons in the hypothalamic arcuate nucleus. In rodents, obesity is associated with leptin resistance due to decreased mRNA expression and translation of its receptor. It is unclear as to why leptin resistance exists in obese humans, although elevated levels seen in overweight individuals may be rendered ineffective by SOCS-3. Exercise reduces leptin levels in proportion to reductions in triglyceride stores in white adipose tissue.
2. Ghrelin is produced by endocrine cells in the gastric mucosa, and exerts orexigenic effects by binding to GHSRs expressed by AgRP/NPY neurons in the hypothalamus and by neurons in the area postrema. Ghrelin also binds to midbrain dopaminergic neurons mediating homeostatic feeding and food-reward behavior, and neurons in the hippocampus and amygdala that mediate more complex behaviors related to food intake. Baseline plasma ghrelin levels are inversely proportion to body weight. High intensity exercise reduces ghrelin levels and energy intake in obese and normal weight subjects.
3. GLP-1 is produced by intestinal L-cells. It reduces appetite by inducing an anorexic phenotype in vagal afferent neurons; GLP-1 expressing neurons in the medulla also send anorexigenic signals to the paraventricular nucleus of the hypothalamus. Plasma GLP-levels are depressed in obese individuals. High intensity exercise causes transient rises in GPL-1 blood levels in both obese and normal weight individuals. GLP-1 and GLP-1 analogues are effective in reducing hunger scores and weight when given to rodents and humans, and GLP-1 is a potent incretin, forming the basis for new treatments of diabetes mellitus.
4. BDNF is widely distributed in the brain. It exerts its anorexigenic effects by binding to TrkB receptors which are highly expressed in POMC/CART neurons, and in neurons in the area postrema, NTS, and dorsal motor nucleus of the vagus. Genetic polymorphisms in BDNF are associated with hyperphagic obesity. Serum BDNF levels may be low in overweight diabetic subjects. High intensity exercise can increase BDNF blood levels.
5. GDF15 is an inflammatory cytokine found in high levels in type 1 macrophages. GDF15 exerts its anorexigenic effects by binding to its receptor GD15AL expressed by POMC/CART neurons and by neurons in the area postrema, and parabrachial and central amygdala nuclei. Vigorous submaximal exercise has been shown to increase GDF15 serum levels in humans.
6. Gut peptide hormones (cholecystokinin, amylin, PP, and PYY) exert their anorexigenic effects in the area postrema, NTS, and dorsal motor nucleus of the vagus. Blood levels of PP and PYY may increase transiently following high intensity exercise.
7. Following bariatric surgery the vagal NTS and vaso-vagal pathways play a major role in restoring normal function to the pancreas (normalizing insulin secretion, reducing glucagon production), liver (recovering insulin sensitivity, reducing gluconeogenesis and free fatty acid release), and gastrointestinal tract (reducing ghrelin secretion, restoring normal responses to nutrients, peptides, hormones).

**Figure 1:**
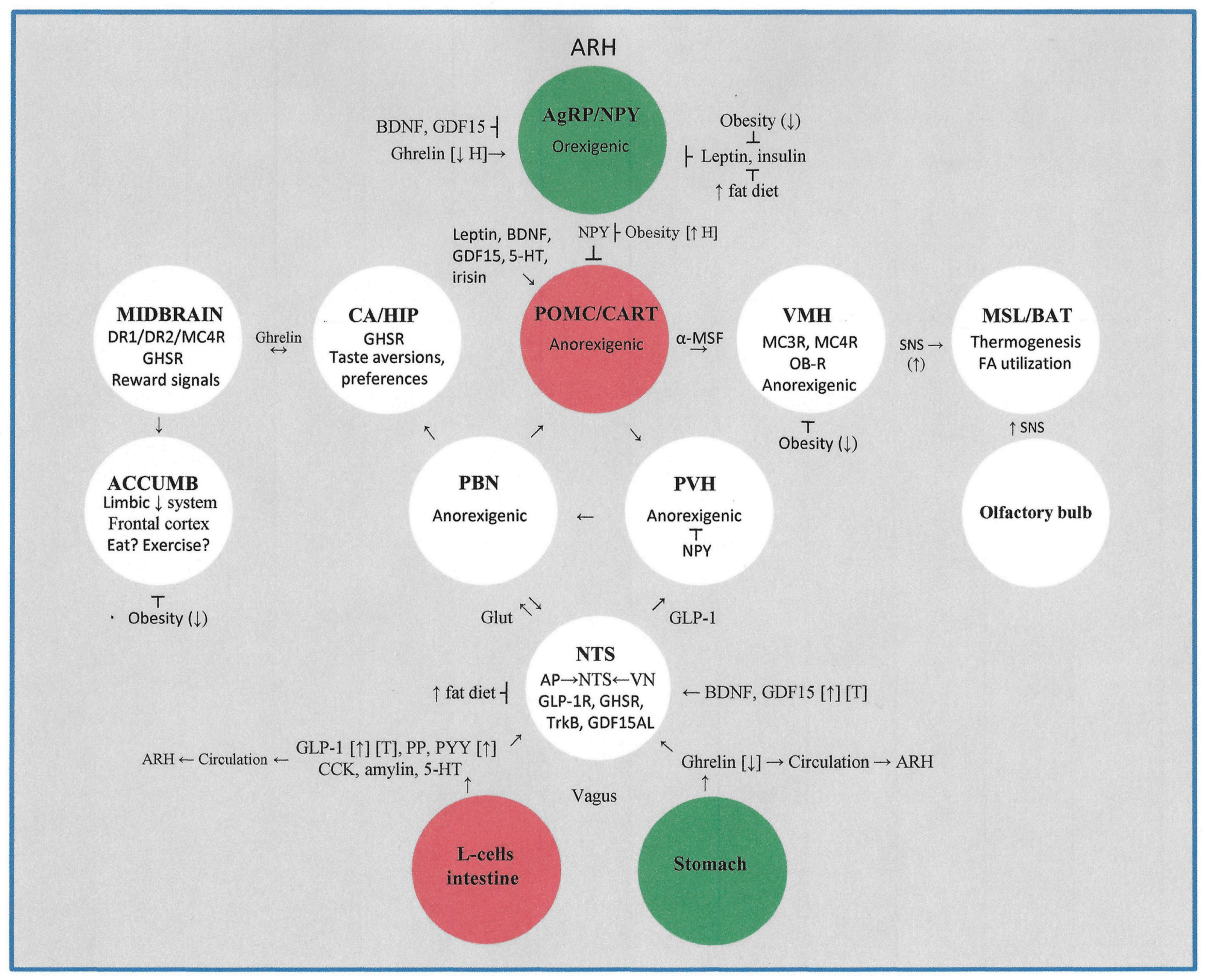
This figure illustrates the major hypothalamic, midbrain, pontine, limbic system, brainstem, and gastrointestinal systems that contribute to the control of energy homeostasis. The impact of obesity, high fat- high energy diets, and moderate to vigorous intensity exercise on energy homeostasis is detailed. Not included is the lateral hypothalamic area which plays an important role in receiving sensory signals from the intestinal tract and liver. ACCUMB, nucleus accumbens; AgRP, agouti-related protein; α-MSF, alpha-melanocortin stimulating factor; AP, area postrema; ARH, arcuate hypothalamus; BAT, brown adipose tissue; BDNF, brain-derived neurotrophic factor; CA, central amygdala; CART, cocaine and amphetamine-regulated transcripts; CCK, cholecystokinin; D1R, dopamine receptor type 1; D2R, dopamine receptor type 2; GDF15, glial-cell-derived growth and differentiation neurotrophic factor 15; GDF15AL, GD15F receptor; GHSR, growth hormone secretagogue receptor (for ghrelin); Glut, glutamine; HIP, hippocampus; MC3R, melanocortin 3 receptor; MC4R, melanocortin 4 receptor; MSL, muscle; NPY, neurotropin Y; NTS, nucleus tractus solitarius; OB-R, leptin receptor; PP, pancreatic peptide; PYY, peptide YY; PBN, parabrachial nucleus; PVH, paraventricular hypothalamus; POMC, pro-opiomelanocortin; SNS, sympathetic nervous system; TrkB, BDNF receptor; VN, vagus nucleus; VMH, ventral medial hypothalamus. Symbols: [↑], exercise increases blood levels; [↓], exercise decreases blood levels; [T], therapeutic potential; (↑H), exercise increases hypothalamic levels; (↓), exercise decreases effect.

Please see Table 1 and Figure 1 for a more comprehensive summary of the effects of obesity, high fat-high energy diets and exercise on the central nervous system control of energy homeostasis. This review does not include the important contributions made by peripheral tissues (e.g., myokines, adipokines other than leptin, and irisin) to energy homeostasis.

**Table 1:** CNS control of metabolic homeostasis.

**Structure**	**Neurons, receptors**	**Effect on energy intake/expenditure**	**Function in obesity or high fat/energy diet**	**Effect of exercise**
HYPOTHALAMUS arcuate & ventromedial nuclei	AgRP/NPY neurons POMC/CART neurons MC3R, MC4R	Orexigenic, ↓ energy expenditure Anorexigenic Anorexigenic, ↑energy expenditure	Resistant to suppression by leptin, insulin; ↓ synapses. Resistant to activation by leptin, suppression by NPY, ↓ neurogenesis MC4R loss causes hyperphagic obesity, insulin resistance	↓ insulin, leptin resistance, ↑ leptin receptor expression, transmission, ↓ghrelin in hypothalamus ↓ leptin resistance, Prevents hyperphagic obesity in young MC4R ^−^/^−^ mice
MIDBRAIN meso-accumbens dopamine system	D1R, D2R, MC4R, GHSR^+^ neurons	Regulate voluntary food intake and energy expenditure (running, locomotion).	↓ energy expenditure due to ↓ D2R+ neuron signaling in nucleus accumbens	Short-term anorexia, ↓weight if energy expenditure > intake
PONS parabrachial nucleus	Calcitonin gene-related peptide^+^ neurons	Primarily anorexigenic (taste aversions, taste preferences).	↓ taste response to sucrose in CCK-1R ^−^/- obese mice	↑ ANS and somatosensory gene expression
MEDULLA area postrema, NTS, & vagus^*^	GDF15R^+^, MC4R^+^, ANS neurons	Orexigenic signals: ghrelin Anorexigenic signals: leptin, CCK, GLP-1, PYY, GDF15, BDNF	Impaired anorexigenic signals & circadian rhythms in NTS	↑ SNS-mediated energy expenditure; ↓ghrelin signaling, ↑GDF15 signaling
DIENCEPHALON olfactory bulb	Olfactory nerves	Anosmia: anorexigenic; ↑energy expenditure Hyperosmia: orexigenic; ↓energy expenditure	Enhanced sense of smell, ↑appetite, ↓energy expenditure	Unknown

* *The vagal nucleus tractus solitarius and vaso-vagal pathways play a preeminent role in the rapid & often persistent remission of type 2 diabetes and the metabolic syndrome in persons undergoing bariatric surgery. AgRP, agouti-related protein; ANS, autonomic nervous system; BDNF, brain-derived neurotrophic factor; CCK, cholecystokinin; CCK-1R, cholecystokinin receptor 1; D1R, dopamine receptor type 1; D2R, dopamine receptor type 2; GDF15, glial-cell-derived growth and differentiation neurotrophic factor 15; MC3R, melanocortin 3 receptor; MC4R, melanocortin 4 receptor; NPY, neurotropin Y; NTS, nucleus tractus solitarius; POMC, pro-opiomelanocortin; SNS, sympathetic nervous system*.

## Discussion

In their review of 16 cohort studies involving 120,813 adults from Europe and the United States, Kivimäki and associates found that the risk of developing cardiometabolic multimorbidity (i.e., having at least two of the following disorders: type 2 diabetes, coronary heart disease, or stroke) was twice as high in overweight individuals (BMI 25.0–29.9 kg/m^2^), nearly five times as high in persons with class 1 obesity (30.0–34.9 kg/m^2^), and nearly fifteen times as high in persons with class 2/3 obesity (BMI > 35.0 kg/m^2^) when compared to normal weight controls. Severely obese individuals were particularly prone to develop diabetes mellitus followed by a vascular event (Kivimäki et al., 2017).

These frightening statistics are a reflection of man's recent success in developing a world rich in hedonic temptations and in technologies that favor indolence over exercise; coupled with the snail's pace of genetic mutations that might allow us to adapt to this world of affluence, our very survival as a species is—in a real sense—dependent on our conscious effort to develop healthful life style changes. What was once a survival benefit (e.g., being able to store energy in fat and tolerate low temperatures by increasing body surface area) has become a survival risk for many of us.

Whereas physical exercise may cause anorexia and weight loss by decreasing plasma levels of ghrelin and/or increasing levels of BDNF, GDF15, GLP-1, and other gut-derived hormones, the effects are usually transient and only associated with weight loss when the exercise is sufficiently taxing to create an energy loss that exceeds energy intake; this may require achieving a VO_2peak_ of at least 60% for a protracted period of time (Broom et al., 2017)—goals seldom achieved in morbidly obese individuals. In obese individuals leptin loses its ability to suppress appetite and increase energy expenditure for reasons that are not fully understood, and midbrain dopamine and limbic centers that normally regulate voluntary motion and food reward signals appear deficient in their ability to control the physical malaise and hedonic eating that characterizes the obese state. On the other hand, GLP-1, a potent anorectic and incretin, has emerged as a promising treatment for both obesity and diabetes mellitus (Näslund et al., 2004). And Blasi has emphasized the importance of the area postrema and the vagal nucleus tractus solitarius in restoring normal function to the pancreas, liver, and gastrointestinal tract following bariatric surgery (Blasi, 2016).; it is no coincidence that this same medullary region plays a major role in transducing orexigenic signals from ghrelin and anorexigenic signals from BDNF, GDF15, and the intestinal hormones GLP-1, cholecystokinin, amylin, PP, and PYY (Wren, 2008; Clemmensen et al., 2017); see Table 1 and Figure 1.

Finally, the full extent of the effect of exercise on the central nervous system is as yet to be determined. Parker and associates measured blood levels of low molecular weight endogenous peptides (the plasma peptidome) in four healthy men undergoing a two-phase exercise regimen: cycling for 6 min at 77% VO_2max_ and then to exhaustion at 87–88% VO_2max_. They found that this exercise program rapidly modulated circulating levels of 425 bioactive peptides through a network of proteases and post-translational modifications, findings that emphasize the extreme complexity of the effects of exercise on the control of metabolic homeostasis (Parker et al., 2017).

## Future directions

Further research is needed to determine the safety and efficacy of anorexigenic peptides such as GLP-1 and GLP-1 analogs, GDF15, and BDNF in inducing weight loss in obese individuals. Trials of exercise training done in low temperature environments, where energy demands are inherently higher, should be assessed. Studies should be done to determine whether exercise training done early in life causes epigenetic changes that have long term beneficial effects on CNS regulation of metabolic homeostasis. Efforts should be made to find medications that can safely downregulate hedonic dietary habits. And, in accordance with World Health Organization recommendations, all school systems should include programs that require children and adolescents to participate in activities that allow them to accumulate at least 60 min of moderate to intense physical activity daily.

## Author contributions

JS is the author of this manuscript and takes sole responsibility for the accuracy of its content.

### Conflict of interest statement

The author declares that the research was conducted in the absence of any commercial or financial relationships that could be construed as a potential conflict of interest.

## Abbreviations

AgRP: agouti-related protein
ANS: autonomic nervous system
BDNF: brain-derived neurotrophic factor
CCK: cholecystokinin
CCK- 1R: cholecystokinin receptor 1
D1R: dopamine receptor type 1
D2R: dopamine receptor type 2
GDF15: glial-cell-derived growth and differentiation neurotrophic factor 15
GLP-1: glucagon-like peptide 1
MC3R: melanocortin 3 receptor
MC4R: melanocortin 4 receptor
NPY: neurotropin Y
NTS: nucleus tractus solitarius
pancreatic peptide: PP
peptide YY: PYY
POMC: pro-opiomelanocortin
SNS: sympathetic nervous system.
